# Daily oscillation of the excitation/inhibition ratio is disrupted in two mouse models of autism

**DOI:** 10.1016/j.isci.2024.111494

**Published:** 2024-12-05

**Authors:** Michelle C.D. Bridi, Nancy Luo, Grace Kim, Benjamin J. Menarchek, Rachel A. Lee, Bryan Rodriguez, Daniel Severin, Cristian Moreno, Altagracia Contreras, Christian Wesselborg, Caroline O’Ferrall, Ruchit Patel, Sarah Bertrand, Sujatha Kannan, Alfredo Kirkwood

**Affiliations:** 1 Zanvyl Krieger Mind/Brain Institute, Johns Hopkins University, Baltimore, MD, USA; 2 Department of Neuroscience, West Virginia University, Morgantown, WV, USA; 3 Department of Anesthesiology and Critical Care Medicine, Johns Hopkins University School of Medicine, Baltimore, MD, USA

**Keywords:** Neuroscience, Sensory neuroscience

## Abstract

Alterations to the excitation/inhibition (E/I) ratio are postulated to underlie behavioral phenotypes in autism spectrum disorder (ASD) patients and mouse models. However, in wild type mice the E/I ratio is not constant, but instead oscillates across the 24-h day. Therefore, we tested whether E/I regulation, rather than the overall E/I ratio, is disrupted in two ASD-related mouse lines: *Fmr1* KO and BTBR, models of syndromic and idiopathic ASD, respectively. The E/I ratio is dysregulated in both models, but in different ways: the oscillation is lost in *Fmr1* KO and reversed in BTBR mice. Phenotypes in both models associate with differences the timing of excitatory and inhibitory synaptic transmission and endocannabinoid signaling compared to wild type mice, but not with altered sleep. These findings raise the possibility that ASD-related phenotypes may be produced by a mismatch between E/I and behavioral state, rather than alterations to overall E/I levels *per se*.

## Highlights

• Altered daily E/I oscillations is *Fmr1* KO and BTBR models of autism
• Absence of E/I oscillations in *Fmr1* KO mice
• Reversed E/I oscillations in BTBR mice
• Autism-associated phenotypes may relate to a mismatch between E/I and behavioral state

## Graphical abstract

**Figure:**
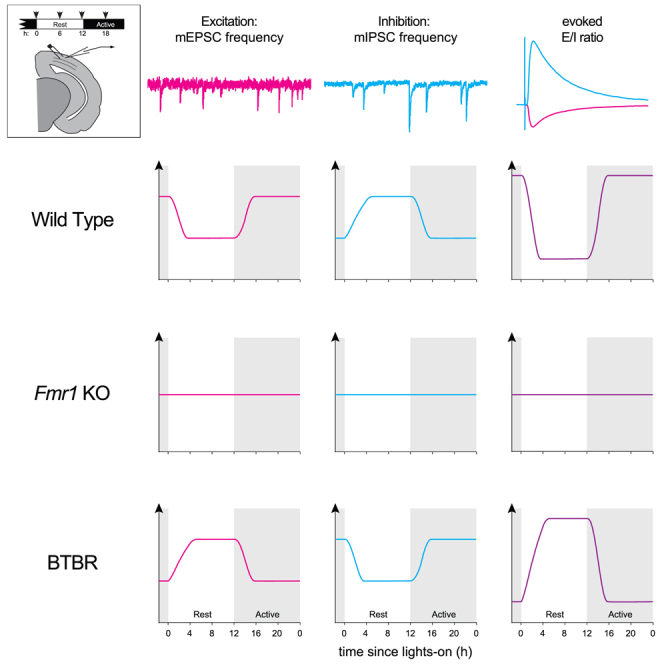

## Introduction

Autism spectrum disorder (ASD) is a prevalent neurodevelopmental disorder estimated to affect 1 in 39 children.^1^ The core features of ASD are language deficits, social interaction deficits, and repetitive or restrictive behaviors and interests. These features are accompanied by sensory abnormalities in >90% of cases, and sensory processing differences have been reported to affect every sensory modality.^2^^,^^3^ The visual system in particular is of great interest, as behavioral deficits have been tied to altered processing in the primary visual cortex (V1) of *Fmr1* knockout (KO) mice.^4^ Moreover, a recent analysis in humans concluded that regarding alterations in gene expression, “the primary visual cortex is the most affected region in ASD”.^5^

ASD is genetically diverse, but elevation of the ratio between excitatory and inhibitory signaling (E/I ratio) in the brain has been proposed as a unifying mechanism.^6^ Accordingly, higher E/I ratio and/or lower inhibition has been observed in sensory cortices of many ASD mouse models.^7^^,^^8^^,^^9^^,^^10^^,^^11^^,^^12^^,^^13^^,^^14^^,^^15^ However, the opposite (decreased E/I ratio, increased inhibition, and/or decreased excitation) has also been reported.^16^^,^^17^^,^^18^^,^^19^^,^^20^^,^^21^

One possible explanation for these opposing findings is that regulation of the E/I ratio, rather than the E/I ratio itself, is disrupted in ASD. In wild type (WT) mice, the E/I ratio in primary visual cortex (V1) changes over the course of the 24-h light:dark cycle, such that it is low during the light (rest) phase.^22^ Therefore, an apparent elevation of the E/I ratio in ASD models could either be due to an increased E/I at all times of day or a dysregulated (e.g., flattened or phase-shifted) E/I oscillation. However, studies that measure the E/I ratio a single time of day cannot distinguish between these possibilities.

These considerations prompted us to examine possible E/I ratio dysregulation in two ASD-related mouse models with disparate genetic causes: the *Fmr1* knockout (*Fmr1* KO) mouse, which models Fragile X syndrome,^23^ the most frequent monogenic cause of intellectual disability and ASD in humans,^24^ and the BTBR *T + Itpr3*^*tf*^*/J* (BTBR) mouse, an inbred line that models idiopathic ASD.^25^^,^^26^ We show that the E/I ratio is dysregulated in both *Fmr1* KO and BTBR mice but in different ways: the oscillation is flattened in *Fmr1* KO mice and the timing of the oscillation is reversed in BTBR mice. Differences in endocannabinoid (eCB) signaling, but not sleep architecture, correspond to the patterns of E/I dysregulation in both lines, suggesting that eCB disruption could be a common feature across genetically diverse forms of ASD.

## Results

### The E/I ratio is dysregulated in two mouse models of ASD

**Figure 1:**
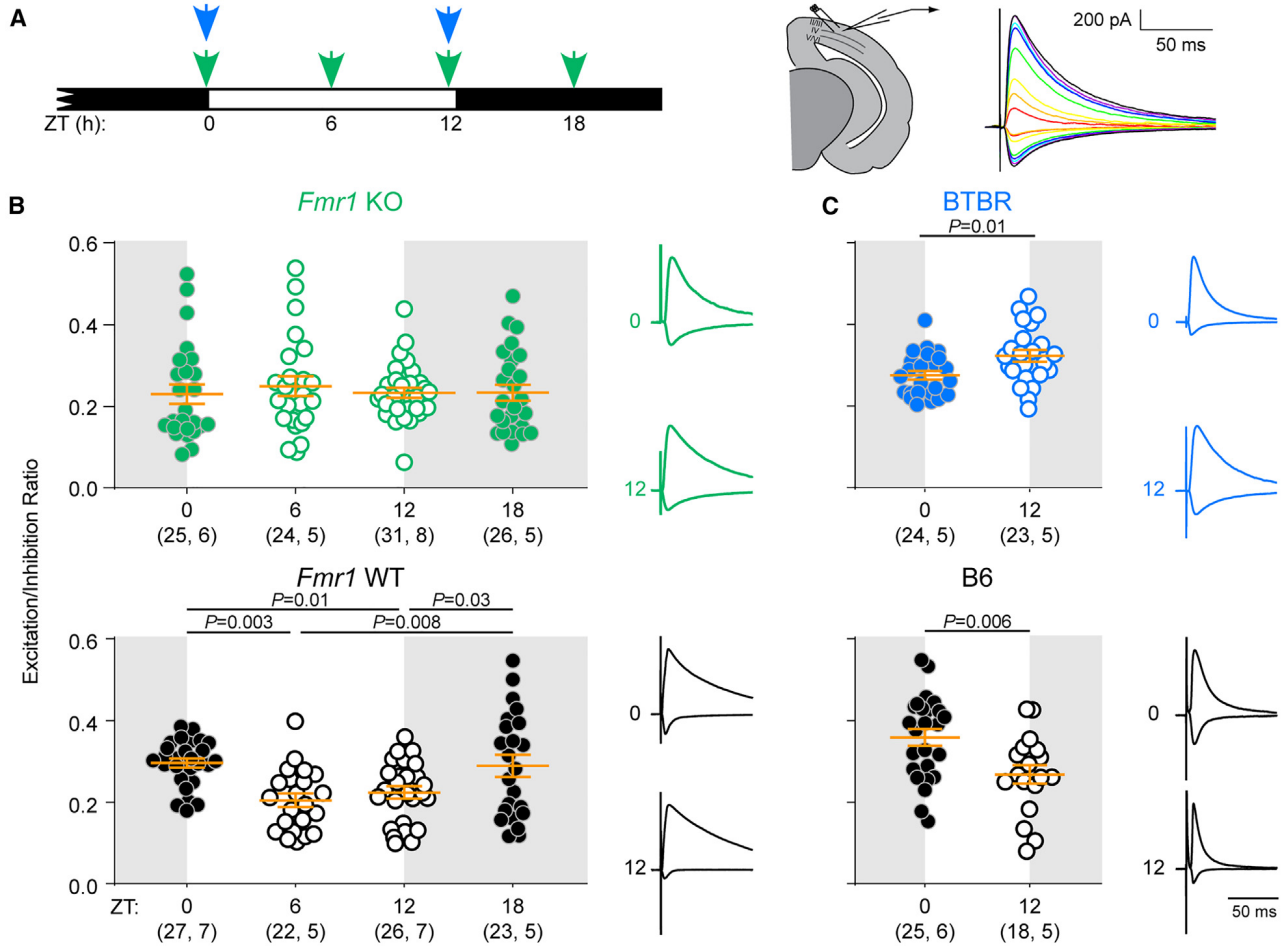
E/I is dysregulated in two mouse models of autism (A) Left: *Fmr1* KO/WT (green) and BTBR/B6 (blue) mice were sacrificed at the indicated times of day. Right: Acute brain slices containing V1 were obtained, and responses to layer 2/3 stimulation over a range of intensities were recorded using whole-cell patch clamp of layer 2/3 pyramidal neurons. Inhibitory (upward deflection) and excitatory (downward deflection) responses were recorded in the same cell and the E/I ratio was calculated using the stimulation intensities over which the E/I ratio was stable.^22^ (B) Oscillation of the E/I ratio across the day is absent in *Fmr1* KO mice. Top: *Fmr1* KO Kruskal-Wallis *p* = 0.65. Bottom: *Fmr1* WT 1-way ANOVA Holm-Sidak post-hoc *p* values indicated. (C) BTBR mice exhibit E/I dysregulation, such that the E/I ratio is high at ZT12, contrasting with higher E/I at ZT0 in B6 mice. *p* values correspond to 2-tailed *t* tests. (B-C) Sample size is indicated as (cells, mice). Error bars indicate mean ± SEM. Example traces are normalized to peak inhibitory response. See also Figure S1. For detailed test statistics, see Tables S1 and S2.

We first determined whether the E/I ratio is dysregulated across the 24-h light/dark cycle in *Fmr1* KO mice. We measured the E/I ratio by electrically stimulating in V1 layer 2/3 and recording excitatory and inhibitory synaptic responses in pyramidal cells lateral to the stimulating electrode (Figure 1A). We initially measured E/I in slices obtained from animals killed at two different time points: ZT0 and ZT12. As expected, in WT controls, the E/I ratio was higher at ZT0 (*t*_(51)_ = 3.92, *p* = 0.0003, unpaired *t* test). In contrast, the E/I ratio in *Fmr1* KO animals was not different between ZT0 and ZT12 (*U* = 312, *p* = 0.22, Mann-Whitney U test), consistent with E/I dysregulation. To distinguish between a flattening vs. altered timing of the E/I oscillation, we then included additional time points (ZT6, 18; Figure 1A). While the E/I ratio in *Fmr1* WT animals was higher when the animal had been in the dark phase (ZT0, 18), there was no time of day effect in *Fmr1* KO animals, indicating that the E/I oscillation is flattened (Figure 1B).

To directly compare between genotypes, we conducted a two-way ANOVA with time of day and genotype as main factors. We previously showed that the E/I ratio becomes stable within 4h of light/dark phase transitions, consistent with our current findings in *Fmr1* WT mice^22^; Figure 1). Therefore, for each genotype, data were pooled into dark (ZT0, 18) and light (ZT6, 12) phase groups. There was a significant interaction between genotype and time of day but no main genotype effect (Table S1), confirming that E/I regulation, rather than overall magnitude, is altered in *Fmr1* KO mice. Post-hoc analysis revealed that the E/I ratio was significantly lower in the dark phase (*p* = 0.003, Holm-Sidak test) but not significantly higher in the light phase (*p* = 0.18) in *Fmr1* KO mice, indicating that the E/I oscillation flattening is predominantly carried by a decrease in the dark phase.

We then examined the E/I oscillation in BTBR and C57Bl/6J (B6) control mice (Figure 1A). As expected, B6 controls exhibited a higher E/I ratio at ZT0 than at ZT12 (Figure 1C). In contrast, the E/I ratio in the BTBR mice followed the opposite pattern, with a higher E/I ratio at ZT12 than ZT0 (Figure 1C). This indicates that the E/I ratio oscillates, but with reversed timing, in BTBR mice. A two-way ANOVA revealed an interaction between genotype and time of day but no main effect of genotype (Table S1), demonstrating that regulation of the E/I oscillation, rather than overall magnitude, is altered in BTBR mice. Post-hoc analysis showed that, compared to B6 controls, BTBR mice had a significantly lower E/I ratio at ZT0 (*p* = 0.001, Holm-Sidak test) and higher E/I ratio at ZT12 (*p* = 0.03), consistent with reversal of the E/I oscillation.

We wished to confirm that V1 circuit function was otherwise normal in these lines. The E/I ratio oscillates over the 24-h day in the lateral (layer 2/3-2/3) circuit, but not in the feedforward (layer 4-2/3) circuit in V1 of WT mice.^22^ To confirm that *Fmr1* KO and BTBR mice did not display an abnormal oscillation in this circuit, we electrically stimulated layer 4 while recording synaptic currents in layer 2/3 of V1 slices. We confirmed that the E/I ratio did not change across the day for *Fmr1* KO, *Fmr1* WT, BTBR, or B6 animals (Figure S1). In contrast with primary somatosensory cortex (S1),^7^ there was no main effect of genotype for either ASD model in the layer 4-2/3 circuit of V1 (Figure S1 and Table S1). Mean ± SEM E/I ratio values for all experiments are presented in Table S2.

Additionally, V1 area is reduced in BTBR mice,^27^ raising the concern that V1 may be fundamentally disorganized, or that visual input may not reach the cortex. Using optical imaging *in vivo,* we confirmed that V1, although smaller, is responsive to visual input and expresses ocular dominance bias and retinotopy comparable to B6 mice (Figure S2). *Fmr1* KO mice are normal in these respects (Figure S2). These results suggest that the E/I ratio phenotypes we observed are not due to a general disruption of circuitry in the visual pathway.

### Altered sleep timing does not explain E/I dysregulation in two mouse models of ASD

Sleep regulates the oscillation of both excitatory and inhibitory synaptic transmission over the course of the day.^22^^,^^28^ Therefore, it is possible that the observed E/I dysregulation in *Fmr1* KO and BTBR mice is due to abnormal sleep timing. Indeed, decreased sleep and altered sleep bout duration and frequency during the light phase have been reported in *Fmr1* KO mice.^29^^,^^30^^,^^31^ We therefore continuously recorded EEG and EMG signals in the home cage using wireless telemetry for three days.

**Figure 2:**
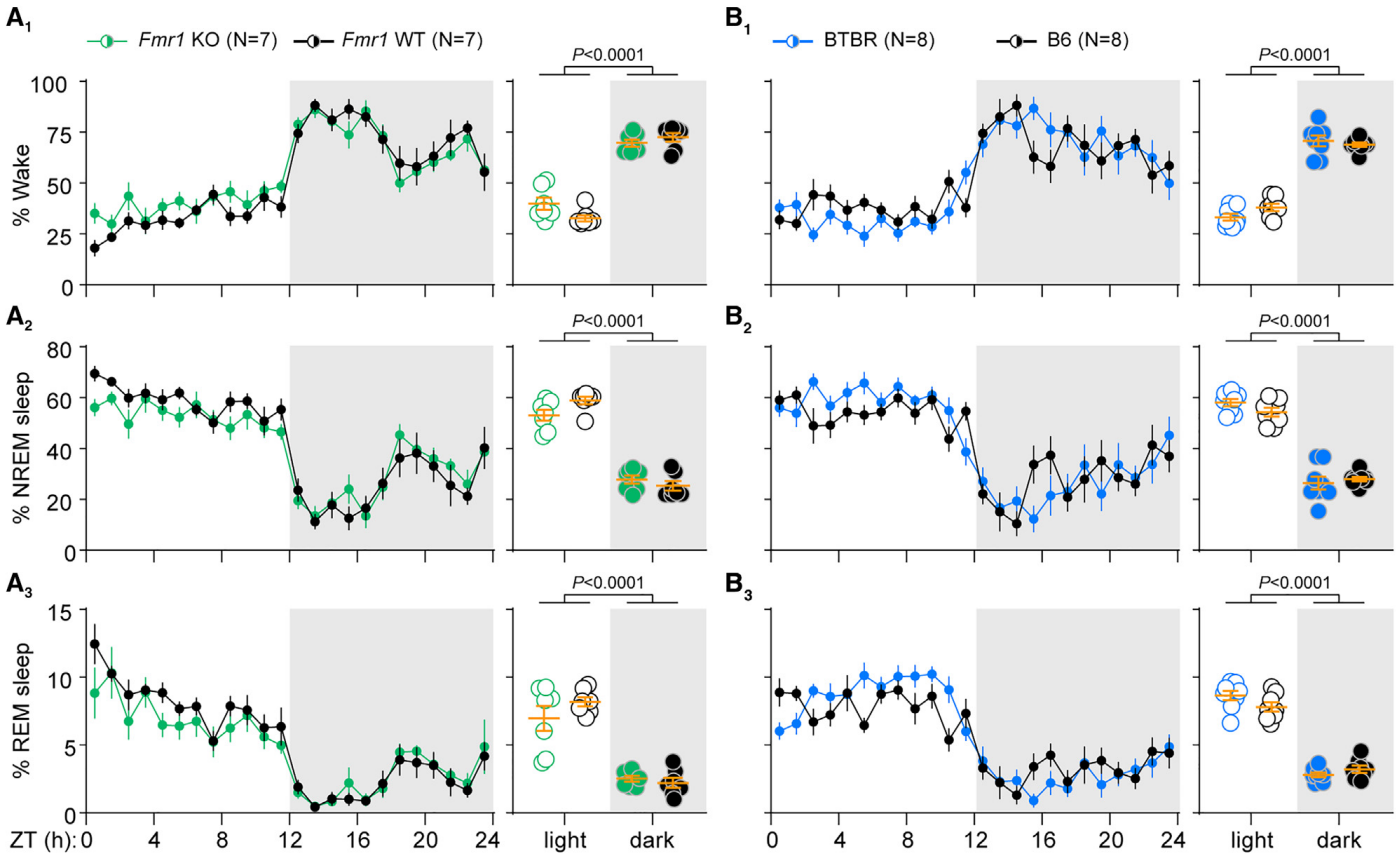
Sleep timing is normal in *Fmr1* KO and BTBR mice (A) Percent time *Fmr1* mice spent awake (A_1_), in NREM sleep (A_2_), and in REM sleep (A_3_). There was no significant main effect of genotype or genotype × time of day interaction for any arousal state when data were broken into 1h bins (left) or averaged across the 12-h light and dark phases (right). There was a significant main time of day effect for all states (∗*p* < 0.05 vs. ZT0, Holm-Sidak post-hoc test). (B) Percent time BTBR and B6 mice spent in each arousal state. BTBR mice displayed grossly normal sleep timing (i.e., slept more during the light phase) and there was no significant main effect of genotype for any arousal state. There were small but significant interaction effects for wake (B_1_) and REM sleep (B_3_) when data were broken into 1h bins (left) and for REM sleep (B_3_) when data were averaged into 12h bins (right). There was a significant main time of day effect for all states (∗*p* < 0.05 vs. ZT0, Holm-Sidak post-hoc test). Data were averaged over 3 recording days for each mouse prior to statistical analysis. All data were analyzed using two-way repeated measures ANOVAs. Data are shown as mean ± SEM. N = number of mice. See also Figure S3. For detailed test statistics, see Table S3.

Sleep timing and architecture was grossly normal in *Fmr1* KO and BTBR mice compared to WT controls. Overall amounts of wake, non-rapid eye movement (NREM) sleep, and REM sleep did not differ between *Fmr1* KO and WT or between BTBR and B6 mice (Figure S3 and Table S3). We did not replicate the NREM or REM sleep deficits reported in *Fmr1* KO mice,^30^^,^^31^ possibly due to an emergence of these deficits with age.^29^ Furthermore, the distribution of time spent in each state across the day did not differ between *Fmr1* KO and WT mice (Figure 2A). The overall pattern of sleep timing appeared normal in BTBR mice, but there was a significant interaction between genotype and time of day for wake and REM amounts (Figure 2B and Table S3). However, these slight differences cannot explain the reversal of the E/I oscillation in BTBR mice, since BTBR mice slept primarily during the light phase.

We also examined the data for evidence of altered sleep quality in each line. Neither *Fmr1* KO nor BTBR mice had fragmented sleep (defined as shorter bout duration, more sleep bouts, and/or more sleep-wake transitions) (Figures S3B–S3E). However, there were slight but significant alterations in spectral power during sleep. Most notably, NREM delta power was decreased in both models and REM theta power was increased in BTBR mice compared to B6 controls (Figure S3E). We also observed a strong trend toward increased waking gamma power in *Fmr1* KO mice (Figure S3F), consistent with previous reports.^31^^,^^32^^,^^33^^,^^34^

### Both excitation and inhibition are dysregulated in two mouse models of ASD

Our findings indicate that the E/I ratio is dysregulated across the day in two ASD-related mouse lines. However, these data do not reveal whether excitation, inhibition, or both are dysregulated. To determine this, we measured miniature excitatory and inhibitory postsynaptic currents (mEPSCs, mIPSCs) in V1 layer 2/3 at ZT0 and ZT12.

**Figure 3:**
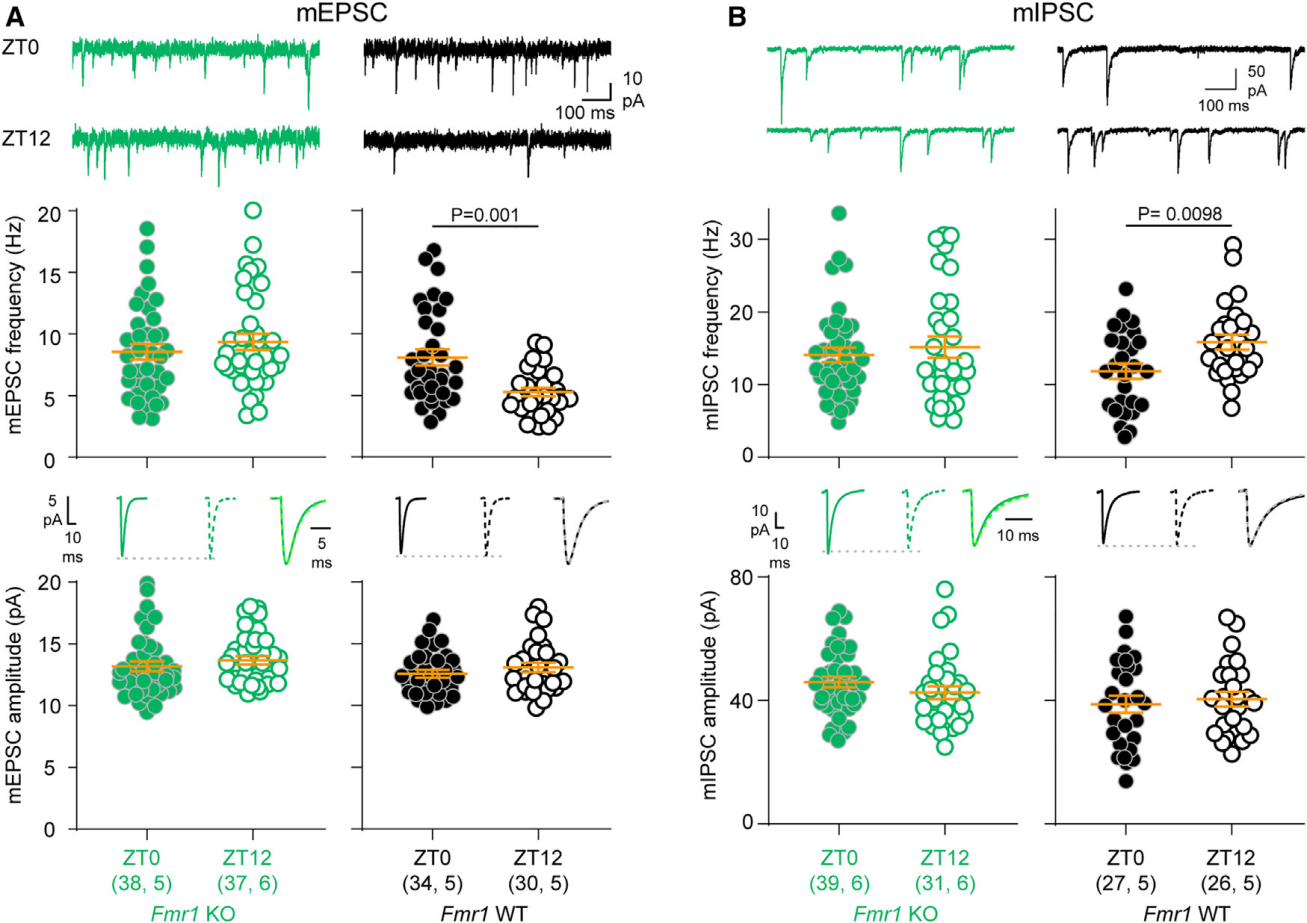
Oscillation of both excitation and inhibition is flattened in *Fmr1* KO mice (A) mEPSC frequency does not change with time of day in *Fmr1* KO mice (*p* = 0.35, Mann-Whitney test), but is higher at ZT0 in *Fmr1* WT control mice (*p* = 0.001, Mann-Whitney test). Amplitude did not change with time of day in either genotype (KO: *p* = 0.19; WT: *p* = 0.39; Mann-Whitney test). (B) mIPSC frequency does not change with time of day in *Fmr1* KO mice (*U* = 596, *p* = 0.93, Mann-Whitney test), but is higher at ZT12 in *Fmr1* WT control mice (*t*_(51)_ = 2.69, *p* = 0.0098, *t* test). Amplitude did not change with time of day in either genotype (KO: *t*_(68)_ = 1.26, *p* = 0.21; WT: *t*_(51)_ = 0.46, *p* = 0.65, *t* test). Data are presented as mean ± SEM. Sample size is indicated as (cells, mice). Averaged traces: solid lines indicate ZT0, dotted lines indicate ZT12; scaled, superimposed averaged traces illustrate that there was no difference in kinetics between the two times of day. For detailed mE/IPSC characteristics and test statistics, see Table S4.

In *Fmr1* WT mice, mEPSC frequency was higher at ZT0 and mIPSC frequency was higher at ZT12 (Figures 3A, 3B, and Table S4). In contrast, mEPSC and mIPSC frequency in *Fmr1* KO mice was the same at both times of day (Figures 3A, 3B, and Table S4). 2-way ANOVAs with genotype and time of day as main factors (Table S1) also revealed a significant main effect of genotype on mEPSC frequency, consistent with an increased density of excitatory synapses in *Fmr1* KO mice.^23^^,^^35^^,^^36^ No time-of-day differences in amplitude were observed for either mEPSCs or mIPSCs (Figure 3 and Table S4).

**Figure 4:**
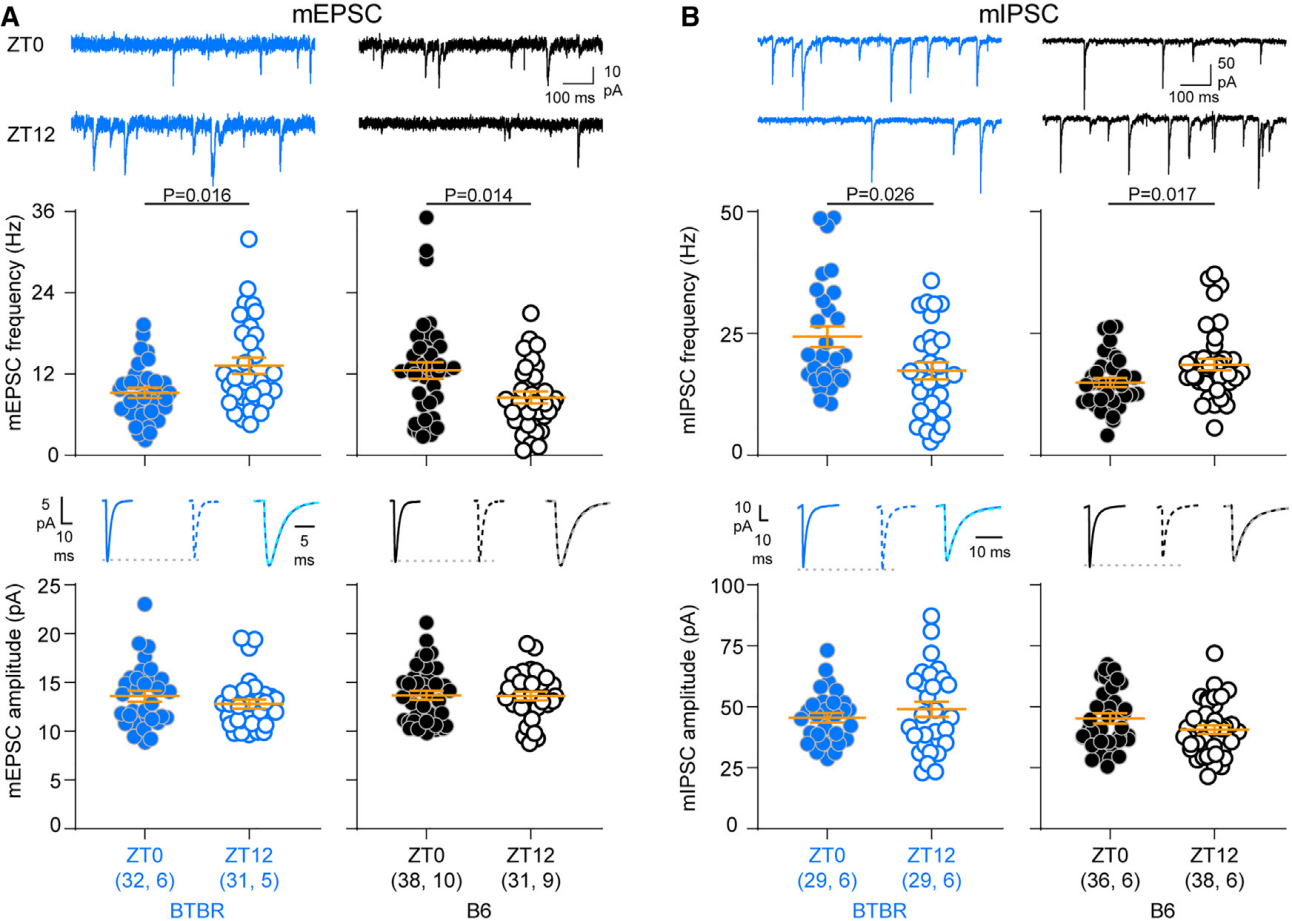
Oscillation of both excitation and inhibition is reversed in BTBR mice (A) mEPSC frequency is higher at ZT12 in BTBR mice (*U* = 322, *p* = 0.016, Mann-Whitney test), and higher at ZT0 in B6 control mice (*t*_(67)_ = 2.53, *p* = 0.014, *t* test). Amplitude did not change with time of day in either genotype (BTBR: *t*_(61)_ = 1.08, *p* = 0.28; B6: *t*_(67)_ = 0.11, *p* = 0.91; *t* test). (B) mIPSC frequency is higher at ZT0 in BTBR mice (*U* = 278, *p* = 0.026, Mann-Whitney test), and higher at ZT12 in B6 controls (*U* = 464, *p* = 0.017, Mann-Whitney test). Amplitude did not change with time of day in either genotype (BTBR: *t*_(56)_ = 0.93, *p* = 0.36; B6: *t*_(72)_ = 1.71, *p* = 0.09, *t* test). Data are presented as mean ± SEM. Sample size is indicated as (cells, mice). Averaged traces: solid lines indicate ZT0, dotted lines indicate ZT12; scaled, superimposed averaged traces illustrate that there was no difference in kinetics between the two times of day. For detailed mE/IPSC characteristics and test statistics, see Table S4.

We performed the same experiment for BTBR and B6 control mice. In BTBR mice, both mEPSC and mIPSC frequency followed a pattern opposite to B6 mice: mEPSC frequency was lower (Figure 4A) and mIPSC frequency was higher (Figure 4B) at ZT0 than at ZT12. 2-way ANOVAs showed significant genotype × time of day interaction effects for both mEPSCs and mIPSCs (Table S1). There were no time-of-day differences in amplitude (Figure 4 and Table S4).

mEPSC and mIPSC frequency track with the patterns of E/I dysregulation in each mouse line (Figure 1), indicating that both excitatory and inhibitory synaptic signaling underlie the dysregulation of the E/I ratio. Differences in mE/IPSC frequency, but not amplitude, across the day are in accordance with our previous study^22^ and are consistent with changes in synapse number and/or presynaptic machinery.

### eCB signaling is dysregulated in Fmr1 KO and BTBR mice

**Figure 5:**
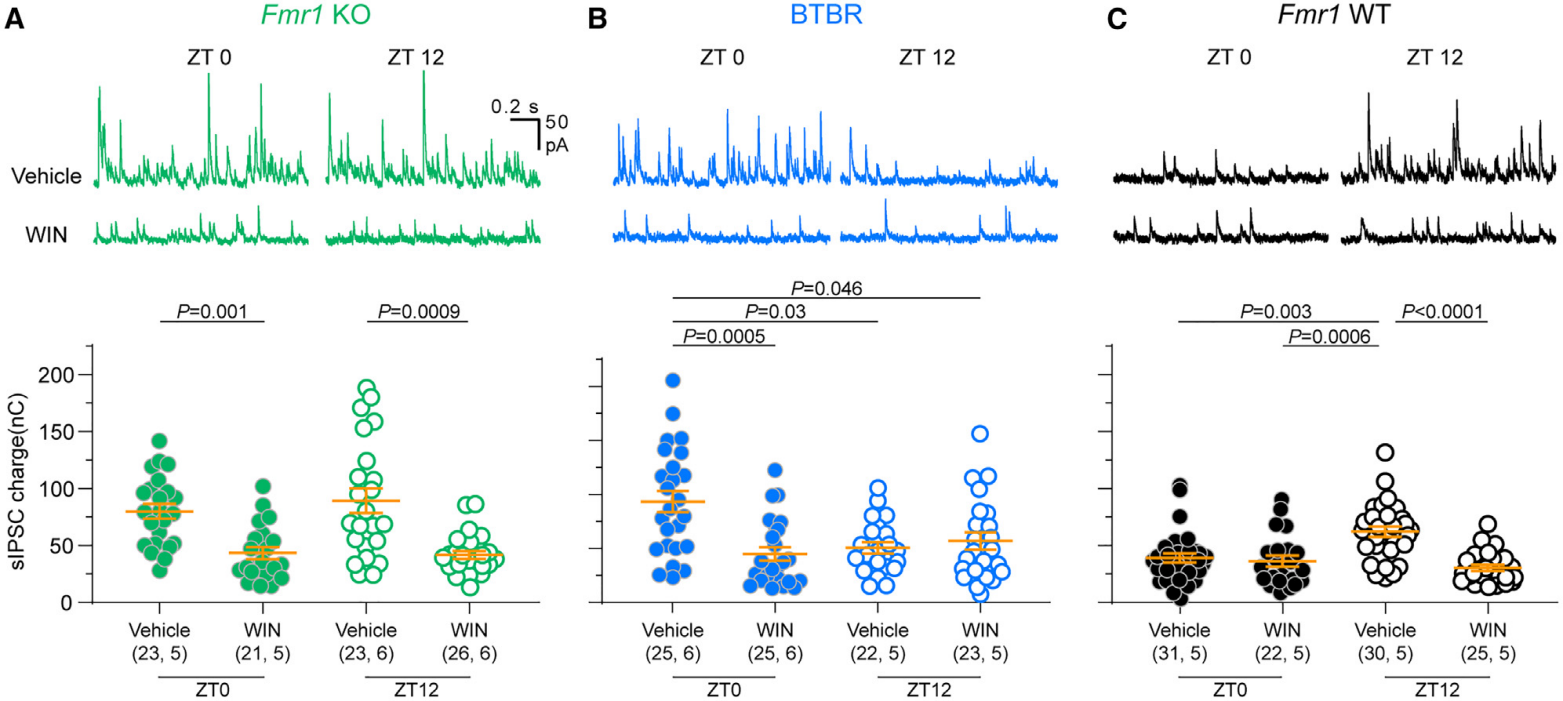
eCB signaling is dysregulated in *Fmr1* KO and BTBR mice Slices were obtained at ZT0 or ZT12 and incubated in the eCB agonist WIN (10μM) or vehicle. sIPSCs were recorded from both treatment conditions in each animal. (A) Example traces (top) and quantification (bottom) of sIPSCs recorded from *Fmr1* KO slices. WIN significantly decreased sIPSC charge at both ZT0 and ZT12. sIPSC charge did not differ between times of day within vehicle or WIN treatment groups (*p* > 0.9999). Kruskal-Wallis ANOVA on ranks *H* = 28.73, *p* < 0.0001. (B) Example traces (top) and quantification (bottom) of sIPSCs recorded from BTBR slices. WIN suppressed inhibitory transmission only at ZT0. Kruskal-Wallis ANOVA on ranks *H* = 16.9, *p* = 0.0007. (C) Example traces (top) and quantification (bottom) of sIPSCs recorded from *Fmr1* WT slices. sIPSC charge was elevated and sensitive to suppression by WIN at ZT12. Kruskal-Wallis ANOVA on ranks *H* = 27.9, *p* < 0.0001. Data are shown as mean ± SEM and sample size is indicated as (cells, mice). *p* values correspond to Dunn’s post-hoc test. See also Figure S4.

**Figure 6:**
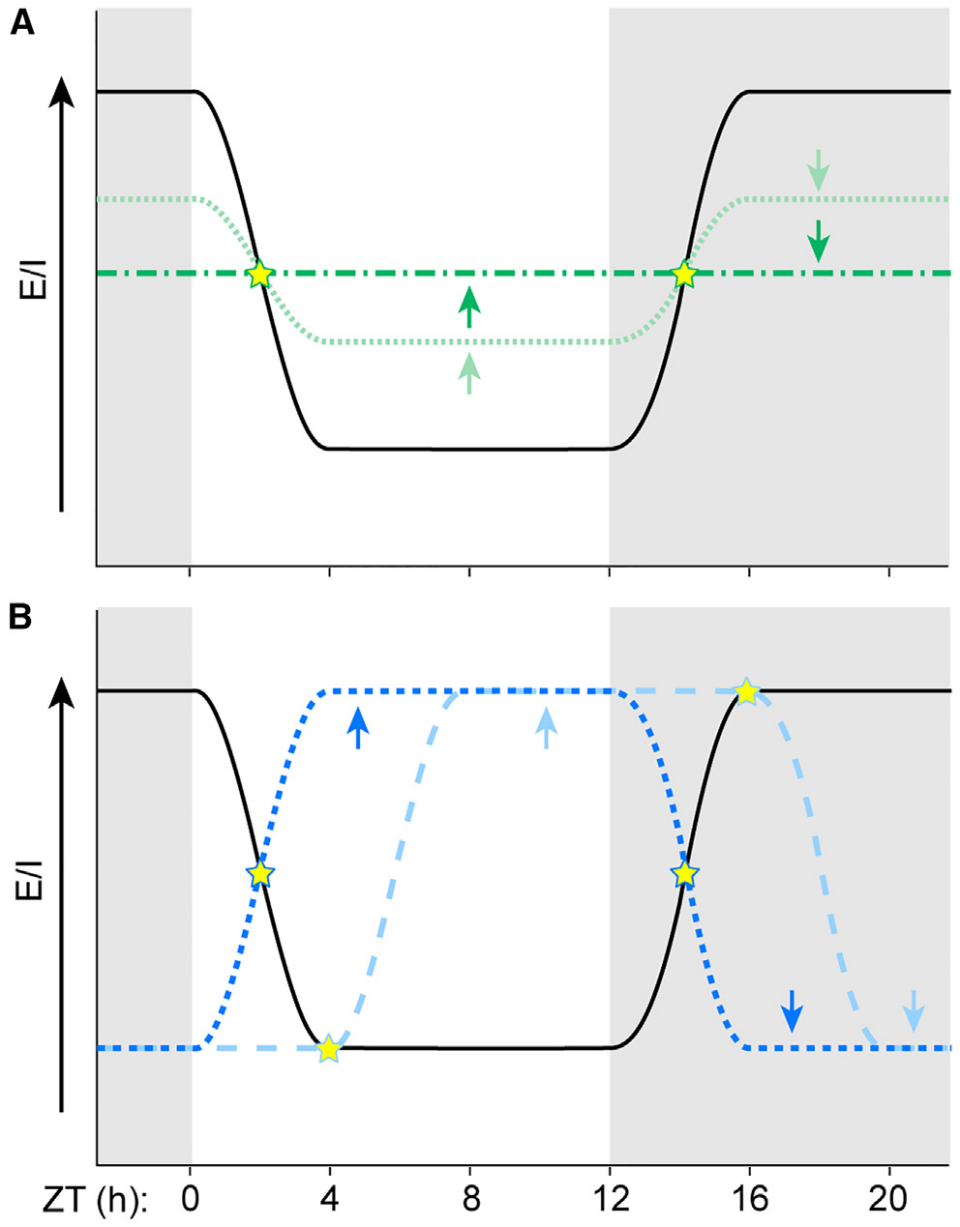
Altered timing or amplitude of the E/I oscillation preserves the WT E/I ratio at multiple times of day (A) Compared to WT (black), decreased amplitude (light green) or complete loss (dark green) of the E/I oscillation causes an apparent increase in E/I ratio during the light phase and/or decrease during the dark phase, but the E/I ratio is not different from WT at two times of day (stars). Our results are consistent with a loss of the E/I oscillation in *Fmr1* KO mice. (B) Compared to WT, altered timing of the E/I oscillation, such as a phase shift (light blue) or phase reversal (dark blue) can cause an apparent increase in the E/I ratio during the light phase, but the E/I ratio is not different from WT at multiple times of day (stars). We observed altered timing of the oscillation in BTBR mice.

Multiple mechanisms can potentially affect the E/I ratio (see discussion). Among them, we previously identified eCB signaling as one candidate mechanism contributing to time-of-day-dependent E/I changes in WT mice.^22^ This raises the possibility that eCB signaling is altered in *Fmr1* KO and BTBR mice. In support of this idea, behavioral phenotypes in both lines can be normalized by pharmacological manipulation of eCB signaling.^37^ Therefore, we tested whether the time-of-day-specific sensitivity of inhibitory transmission to the eCB receptor agonist (+)-WIN 55,212-2 (WIN) is altered in each line. As previously, we evaluated the effects of WIN on spontaneous inhibitory currents (sIPSCs) because under standard slice recording conditions the agonist only affects GABA responses evoked by action potentials,^38^ not mIPSCs. In WT mice, WIN suppresses sIPSCs when endogenous eCB levels are low (ZT12), and this effect is occluded when endogenous eCB levels are high (ZT0) (Bridi et al., 2020; Figure 5). In contrast, both *Fmr1* KO and BTBR mice showed altered patterns of WIN sensitivity. In *Fmr1* KO mice, WIN reduced sIPSCs at both times of day (Figure 5A). In BTBR mice, WIN suppressed sIPSCs when inhibition is abnormally high (ZT0) but this effect was occluded when inhibition was already low (ZT12) (Figure 5B). Repeating this experiment using an eCB receptor antagonist (SR141716A, SR) yielded complementary results (Figure S4). Together, these results are consistent with flattening and reversal of the evoked E/I balance in *Fmr1* KO and BTBR mice, respectively, that is driven in part by altered eCB signaling (Figure 6). We also note that additional mechanisms might regulate the daily modulation of mIPSCs.

## Discussion

In WT mice, the E/I ratio, correlations between ionic conductances, and membrane capacitance vary over the 24-h day, indicating daily rearrangement of connectivity and function under normal conditions.^22^^,^^39^^,^^40^^,^^41^ Therefore, it is crucial to consider neuronal dysfunction in models of neurological conditions against a backdrop of dynamic circuit properties. In this study, we show that the daily E/I oscillation is dysregulated due to changes in both excitatory and inhibitory synaptic transmission in two mouse lines associated with ASD. The oscillation is flattened in *Fmr1* KO, and reversed, consistent with altered phase and/or periodicity, in BTBR mice. These changes cannot be explained by sleep timing, but patterns of inhibition are consistent with altered eCB signaling. Exploring how this dysregulation occurs is an important avenue for future investigation.

### Implications for ASD

A prominent hypothesis in the field states that elevated E/I ratio is a common mechanism leading to the behavioral phenotypes across genetically diverse forms of ASD.^6^ We propose to refine this viewpoint: on average, the magnitude of the E/I ratio is normal, but it is elevated/lowered at inappropriate times of day (Figure 6). This may result in a mismatch between E/I and brain state that affects behavioral phenotypes.

While the prevailing view suggests that the E/I ratio is elevated in models of ASD, findings regarding inhibition are often mixed, with some studies reporting increased inhibition or no changes at all. This is evident even within the same genetic models, such as the CNTNAP2^−/−^ model.^42^^,^^43^^,^^44^ Identifying the patterns of E/I dysregulation specific to each ASD-related mouse line opens a new perspective that may reconcile discrepancies across studies. The E/I ratio is low during the light (rest) phase in WT animals.^22^ Therefore, measuring the E/I ratio exclusively during this phase may lead studies to inaccurately report an elevated E/I ratio in ASD mouse lines, despite an absence of overall differences (Figure 6). Similarly, measurements done at different phases of the light/dark cycle might yield different values in the same ASD mouse lines. Supporting the notion of dysregulation, lower mIPSC frequency has been reported in V1 L2/3 of *Cntnap2*-, *Tsc1-,* and *Ube3a-*deficient mice.^9^^,^^13^^,^^15^ In contrast, *Fmr1* KO mice have normal mIPSC frequency and spontaneous activity in V1 L2/3^4^^,^^45^, which may be attributable to differences in the time of day at which recordings were conducted and/or genetic background.

In this study, we examined V1 because the E/I ratio oscillation in WT mice is best characterized in this region. However, the E/I ratio also undergoes a daily oscillation in hippocampus and medial prefrontal cortex,^22^ raising the possibility that E/I dysregulation may extend to other brain regions. Consistent with this idea, the E/I ratio is elevated in CA1 of *Fmr1* KO and BTBR mice,^46^^,^^47^^,^^48^^,^^49^ but whether these findings are due to dysregulation remains to be seen. Regardless, our positive results in V1 confirmed our central hypothesis of E/I dysregulation.

Furthermore, the E/I ratio oscillation is specific to the layer 2/3-2/3, but not the layer 4-2/3, pathway in WT mice (Figure S1; see Bridi et al.^22^). Here, we show that the E/I ratio remains fixed in the layer 4-2/3 circuit of both *Fmr1* KO and BTBR mice. Notably, the E/I ratio was not elevated in this circuit in either line, suggesting that pathways in which the E/I ratio is dynamically regulated are more likely to be affected in ASD models. However, the E/I ratio is elevated in S1 layer 4-2/3 of *Fmr1* KO mice.^7^ While differences in genetic background and age between the two studies may explain this discrepancy, these findings also raise the possibility that circuit-specificity of the E/I ratio oscillation may differ across cortical regions.

### Candidate mechanisms of E/I dysregulation

One intriguing implication of our findings is that regulation of excitatory and inhibitory synaptic transmission go hand-in-hand, since they are jointly dysregulated in both *Fmr1* KO and BTBR mice. This may occur via a central mechanism controlling both excitation and inhibition, or by the dependence of excitation on inhibition (or vice versa). While the exact mechanisms remain to be elucidated, changes in mE/IPSC frequency are consistent with formation/elimination of synapses across the 24-h day, implicating a number of potential candidate mechanisms.

Four moving parts comprise the E/I ratio oscillation in WT mice: increased excitation/decreased inhibition during the active phase, and decreased excitation/increased inhibition during the rest phase. During the active phase, long-term potentiation has been implicated in increased excitatory transmission, while eCB signaling, visual experience, and cholinergic signaling control decreased inhibitory transmission. During the rest phase, sleep is required for decreased excitation and increased inhibition.^22^^,^^28^^,^^40^^,^^50^ The ways in which these mechanisms operate in WT mice remain poorly understood, limiting our ability to interpret the exact mechanisms of dysregulation in *Fmr1* KO and BTBR mice. Nevertheless, we examined how two of these mechanisms, sleep and eCB signaling, relate to E/I dysregulation.

Sleep disruption is a common feature of ASD.^51^ However, despite a variety of altered sleep and activity characteristics, a grossly normal nocturnal activity pattern is preserved across many commonly used ASD-related mouse lines.^29^^,^^31^^,^^52^^,^^53^^,^^54^^,^^55^^,^^56^^,^^57^^,^^58^^,^^59^^,^^60^^,^^61^^,^^62^^,^^63^^,^^64^^,^^65^^,^^66^ Here, we confirmed that sleep timing follows a normal pattern of nocturnal activity in *Fmr1* KO and BTBR mice. Therefore, dysregulation is unlikely to arise from altered sleep timing. This suggests that sleep/wake states may be uncoupled from E/I regulation in *Fmr1* KO and BTBR mice, pointing to a failure of sleep function in these lines. However, NREM delta power was reduced in both genotypes, consistent with reports in other mouse lines,^52^^,^^53^^,^^60^^,^^61^^,^^63^^,^^67^ and altered neuronal activity during NREM sleep could contribute to E/I dysregulation.

eCB signaling has also been implicated in ASD in humans,^68^^,^^69^ as well as in *Fmr1* KO, BTBR, and other ASD-related mouse lines.^37^^,^^70^^,^^71^^,^^72^^,^^73^^,^^74^^,^^75^^,^^76^^,^^77^ Here, we report that inhibitory transmission is susceptible to manipulations of eCB signaling in *Fmr1* KO and BTBR mice, indicating that eCB signaling pathways are operational. However, the response to eCB manipulation is flattened in *Fmr1* KO and reversed in BTBR mice, consistent with altered timing of eCB release and/or eCB receptor expression contributing to dysregulation of inhibition in both lines.

An additional candidate mechanism is glial function. Microglia and astrocytes play a role in pruning and function of excitatory and inhibitory synapses,^78^^,^^79^^,^^80^^,^^81^^,^^82^^,^^83^^,^^84^ thus shaping connectivity in the cortex.^85^ Importantly, microglial and astrocyte function, which in WT mice depend on arousal state,^86^^,^^87^^,^^88^^,^^89^^,^^90^^,^^91^ is altered in both *Fmr1* KO and mice BTBR mice.^92^^,^^93^^,^^94^^,^^95^^,^^96^ Whether glia play a role in E/I dysregulation, however, remains to be explored.

### Consequences of dysregulated E/I balance

E/I fluctuations in V1 L2/3 across the 24-h day in WT mice alter circuit excitability *ex vivo* and pyramidal cell activity *in vivo*,^22^^,^^40^ and have the potential to modulate visual processing properties such as orientation tuning (reviewed in Isaacson and Scanziani^97^). However, the exact behavioral impact of altered E/I balance in ASD remains largely unclear. A common view states that network hyperexcitability associated with reduced inhibition and altered inhibitory plasticity impairs neural processing (for recent reviews see^98^^,^^99^^,^^100^^,^^101^), an idea well suited to account for perceptual learning deficits resulting from impairments in sensory discrimination.^4^ Alternatively, altered excitatory and inhibitory synaptic transmission may not be a primary effect of the ASD phenotype, but rather reflect homeostatic compensations that mitigate other types of dysfunction.^7^^,^^102^^,^^103^ E/I dysregulation might offer unique opportunities to discriminate between these two possibilities because E/I differences between mutant and WT animals will vary in magnitude and sign across the day. In these cases, time-of-day dependent variations in the degree of behavioral impairments, as reported recently,^104^ correlating with daily E/I discrepancies will support a direct impact of altered E/I on behavioral phenotype.

### Conclusions

The E/I ratio is dynamically regulated, likely via multiple mechanisms acting in concert. Each of these mechanisms has the potential to be affected by a variety of genetic and environmental factors, making the E/I oscillation vulnerable to disruption. Therefore, E/I dysregulation may be a point of convergence for genetically diverse forms of ASD, ultimately resulting in common behavioral phenotypes. Further exploring the underlying mechanisms will provide crucial insight to the pathophysiology of ASD.

### Limitations of the study

Altered E/I ratio is widely considered a unifying hypothesis in the ASD field. We show that alterations in the phase and/or amplitude of daily oscillations of the E/I ratio are better descriptors in V1 of two autism-related mouse lines. It remains unclear whether these findings generalize to other brain regions, other genetically diverse mouse models of autism, and whether they occur in humans. Furthermore, the mechanisms that regulate daily E/I ratio fluctuations in WT mice are incompletely understood. Here, we investigated two mechanisms identified in WT mice, sleep, and eCB signaling; however, a more complete understanding of how these mechanisms operate and interact in WT mice is needed in order to determine how E/I dysregulation arises in ASD models. It is also unclear how regulation of the E/I ratio over the 24-h day impacts sensory processing and behavior in WT mice, limiting our understanding of how behavioral phenotypes may be impacted by E/I dysregulation in ASD models.

## Resource availability

### Lead contact

Requests for further information and resources should be directed to and will be fulfilled by the lead contact, Alfredo Kirkwood (elkirkwood@gmail.com).

### Materials availability

This study did not generate new unique reagents.

### Data and code availability

• Data: Raw data have been deposited to Open Science Framework and are publicly available as of the date of publication. The DOI is listed in the key resources table.
• Code: Original code has been deposited at GitHub and is publicly available as of the date of this publication. The URL is listed in the key resources table.
• Additional information: Any additional information required to reanalyze the data reported in this paper is available from the lead contact upon request

## Acknowledgments

AK is supported by 10.13039/100000053NEI grants R01EY12124 and R01EY025922. MB is supported by NIH NIGMS P20GM109098, NSF 2242771, 10.13039/100000874Brain & Behavior Research Foundation 31841. BJM is supported by NIH NIGMS T32GM132494, and BR is supported by 10.13039/100005768NASA West Virginia Space Grant Consortium, Grant # 80NSSC20M0055.

## Author contributions

M.C.D.B. conceptualized and performed experiments, analyzed data, and wrote the manuscript. N.L., G.K., B.J.M., R.A.L., B.R., D.S., and C.W. performed experiments and analyzed data. S.B., R.P., and C.O. assisted with data collection. S.K. supervised experiments and reviewed the manuscript. A.K. conceptualized and supervised experiments and wrote the manuscript.

## Declaration of interests

The authors declare no competing interests.

## STAR★Methods

### Key resources table

**Table:** 

REAGENT or RESOURCE	SOURCE	IDENTIFIER
**Chemicals, peptides, and recombinant proteins**		

DL-AP5 (APV)	Tocris	Cat # 0105
Tetrodotoxin Citrate (TTX)	Abcam	Cat # ab120055
CNQX disodium salt hydrate	Sigma-Aldrich	Cat #C239
SR-95531 (Gabazine)	Sigma-Aldrich	Cat #S106
(+)-WIN55,212-2 mesylate	Cayman Chemical	Cat # 10009023
SR141716A (Rimonabant Hydrochloride)	Sigma-Aldrich	Cat # SML0800

**Experimental models: Organisms/strains**		

Mouse: C57BL/6J (B6)	Jackson Laboratory	Cat #000664; RRID: IMSR_JAX:000664
Mouse: B6.129P2-*Fmr1*^*tm1Cgr*^/J (*Fmr1* KO)	Jackson Laboratory	Cat #003025; RRID: IMSR_JAX:003025
Mouse: BTBR *T*^*+*^*Itpr3*^*tf*^/J (BTBR)	Jackson Laboratory	Cat # #002282; RRID:IMSR_JAX:002282

**Software and algorithms**		

Igor Pro	WaveMetrics, Inc	RRID:SCR_000325
SutterPatch	Sutter Instruments	N/A
MiniAnalysis	BlueCell	RRID:SCR_002184
MATLAB	MathWorks	RRID:SCR_001622
sIPSC analysis code for MATLAB	Bridi et al. 2020^22^	https://github.com/michellebridi/sISPC
Ponemah	Data Sciences International	RRID:SCR_017107
SleepSign for Animal	Kissei Comtec, Ltd.	RRID:SCR_018200
Prism	GraphPad	RRID:SCR_002798

**Other**		

Whole-cell patch clamp, sleep recording, and optical imaging data	This publication	https://doi.org/10.17605/OSF.IO/9KWUH

### Experimental model and study participant details

All procedures were approved by the Johns Hopkins University (approval # MO20M278) and/or West Virginia University (approval # 2210059267) Institutional Animal Care and Use Committee. Mice were bred in-house on a 12:12 light:dark cycle (lights on at 6a.m. or 7a.m.). Upon weaning (postnatal day (P) 21), naive mice were group housed and entrained for at least two weeks to a 12:12 light:dark cycle in custom entrainment chambers, with the timing of lights-on adjusted according to the experiment. Due to X-linkage of the *Fmr1* gene, only male *Fmr1* WT and KO mice (Jackson Laboratory #003025) were used. *Fmr1* KO mice were backcrossed to C57Bl/6J mice to generate *Fmr1* heterozygous females, which were then backcrossed again to C57Bl/6J mice to produce WT and KO littermates. C57Bl/6J (Jackson laboratory #000664) and BTBR (Jackson Laboratory #002282) mice of either sex were used. Littermates were distributed across experimental groups. At the time of all experiments, mice were between P35 and P56.

### Method details

#### Slice preparation & whole cell recording

Animals were killed within a 30-min window prior to the indicated ZT. 300 μm thick coronal brain slices containing V1 were prepared as described previously.^22^ Briefly, slices were cut in ice-cold dissection buffer containing 212.7 mM sucrose, 5 mM KCl, 1.25 mM NaH_2_PO_4_, 10 mM MgCl_2_, 0.5 mM CaCl_2_, 26 mM NaHCO_3_, and 10 mM dextrose, bubbled with 95% O_2_/5% CO_2_ (pH 7.4). Slices were transferred to normal artificial cerebrospinal fluid (similar to the dissection buffer except that sucrose was replaced by 119 mM NaCl, MgCl_2_ was lowered to 1 mM, and CaCl_2_ was raised to 2 mM) and incubated at 30°C for 30 min and then at room temperature for at least 30 min before recording.

Visualized whole-cell voltage-clamp recordings were made from pyramidal neurons in L2/3 (35% depth from the pia) of V1. In slices from BTBR mice, recordings were restricted to a smaller posterior/medial region to account for the size and location of V1 in these mice.^27^ Glass pipette recording electrodes (3–6 MΩ) were filled with different internal solutions according to each experiment, all of which were adjusted to pH 7.2–7.3, 280–295 mOsm. Cells with an input resistance ≥150 MΩ and access resistance ≤25 MΩ were recorded. For all whole cell recordings, cells were discarded if these values changed more than 25% during the experiment. Data were filtered at 2 kHz and digitized at 10 kHz using Igor Pro (WaveMetrics, Portland, OR) and SutterPatch (Novato, CA) software.

For E/I ratio recordings, the internal pipette solution for recording evoked EPSCs and IPSCs contained 8mM KCl, 125 mM cesium gluconate, 10 mM HEPES, 1 mM EGTA, 4mM Mg-ATP, 0.5mM Na-GTP, and 5 mM QX-314. Responses were recorded in the presence of 100 μM DL-APV. Reversal potentials for excitatory and inhibitory currents of +10 mV and −55 mV (without junction potential compensation) were used.^22^ To evoke synaptic responses, a double-barrel glass stimulating pipette filled with ACSF was placed approximately 100–200 μm lateral to the recording electrode (layer 2/3 stimulation) or in the middle of the cortical thickness (layer 4 stimulation). A series of stimulations over a range of intensities was delivered, and the responses over the range of intensities producing a stable E/I ratio were used.^22^

For mEPSC recordings, 1 μM TTX, 100 μM DL-APV and 2.5 μM gabazine were added to the perfusion buffer to isolate AMPAR-mediated mEPSCs. An internal pipette solution containing the following ingredients was used: 8 mM KCl, 125 mM cesium gluconate, 10 mM HEPES, 1 mM EGTA, 4 mM NaATP, and 5 mM QX-314. To record mIPSCs, 1 μM TTX, 100 μM DL-APV and 20 μM CNQX were included in the bath. The internal pipette solution contained: 120 mM CsCl, 8 mM KCl, 10 mM EGTA, 10 mM HEPES, and 5 mM QX314. V_m_ was held at −70 mV.

To record sIPSCs, conditions were similar to mEPSC recording except no QX-314 was included in the internal pipette solution, V_m_ was held at +10 mV, and the bath contained only 10μM (+)-WIN55,212-2 (Cayman Chemical, Ann Arbor, MI), 10μM SR 141716A (Sigma-Aldrich, St. Louis, MO), or vehicle (0.1% DMSO). Control and drug-treated slices were obtained from the same animals.

#### Polysomnography recording

4-5 week old mice were placed under isoflurane (1–2%) anesthesia and immobilized. A pocket was formed under the skin and a wireless transponder attached to four recording leads (model HD-X02, Data Sciences International, St. Paul, MN) was inserted into the pocket. Two EMG leads with 0.5 cm exposed wire were inserted into the cervical trapezius muscles and held in place with 5-0 silk sutures. The skull was cleaned with H_2_O_2_ and two holes were drilled for EEG lead placement (location posterior to bregma/lateral to the midline: 3.4mm/2.5mm and 1mm/1mm). EEG leads with 1–2 mm exposed wire were inserted into the holes to make contact with the dura and affixed with dental acrylic and cyanoacrylate glue. The wound was sutured closed and treated with triple antibiotic ointment. Mice were allowed to recover in their home cage for at least 7 days before recording. Home cages were then placed on top of telemetry receiver pads and EEG and EMG signals were captured (Data Sciences International, PhysioTel Receiver RPC-1) using Ponemah software (Data Sciences International, Version 6.40) for 3 consecutive days on a light-dark cycle matching the vivarium environment. The EEG/EMG signal was sampled at 500 Hz.

#### Optical imaging of the intrinsic cortical signal

Animals were anesthetized using isoflurane in O_2_ (induction: 2–3%, maintenance: 0.5–1%) supplemented with chlorprothixene (2 mg/kg i.p.). An incision was made in the scalp and lidocaine was applied to the margins. Exposed skull above V1 was covered in 3% agarose and an 8mm round glass coverslip. Surface vasculature was visualized by illuminating the area with 555nm light. Then the camera was focused 600 μm below the cortical surface and the area was illuminated with 610 nm light. A high refresh rate monitor (1024 × 768 @120 Hz; ViewSonic, Brea, CA) was aligned in the center of the mouse’s visual field 25cm in front of the eyes. Visual stimuli consisted of a white horizontal bar on a black background (2° height) presented either to the entire visual field or only to the binocular visual field (−5° to +15° azimuth), moving continuously upward or downward (5 min per direction). Cortical signals were imaged using a Dalsa 1M30 CCD camera (Dalsa, Waterloo, Canada). Optical imaging was always performed during the light phase.

### Quantification and statistical analysis

#### Whole-cell recordings

To quantify the E/I ratio, peak response amplitude at each holding potential was measured for each stimulus intensity and the ratio between the excitatory and inhibitory peak was calculated (Igor Pro). If multiple peaks were observed in the postsynaptic response, the magnitude of the first peak was used in order to limit the analysis to monosynaptic responses. If the first peak could not be clearly resolved, the cell was discarded from the analysis.

mEPSCs and mIPSCs were analyzed using the MiniAnalysis program (Synaptosoft, Decatur, GA). Only cells with root-mean-square (RMS) noise <2 (mEPSCs) or <4 (mIPSCs) were included in the analysis and event detection threshold was set at 3 times the RMS noise. 300 events with rise time <3 msec (mEPSCs) or <5 msec (mIPSCs) were selected for each cell to calculate frequency and amplitude. Non-overlapping events were used to construct the averaged traces.

Spontaneous IPSCs were analyzed by calculating the unit charge (nA/s) with custom code (MATLAB, MathWorks, Inc, Natick, MA).^22^ The baseline was calculated and subtracted for each 500 msec of recording. Charge was calculated as the integral of the baseline-subtracted signal. 3–4 min of recording were quantified for each cell.

#### Polysomnography

Arousal stages were scored manually offline as NREM sleep, REM sleep, and wake by a trained experimenter (MB) in 4-s epochs (SleepSign for Animal, Kissei Comtec). The percent time spent in each state, along with number and duration of bouts, was calculated. Power spectra were computed within each arousal state by performing a fast Fourier transform on the EEG signal with 0.5 Hz resolution. For each arousal state, spectra were normalized to the total EEG power (0.5–80 Hz) in that state. One B6 and one BTBR animal were included in sleep architecture analysis but excluded from spectral analysis due to differences in electrode placement.

#### Optical imaging of the intrinsic cortical signal

The cortical response at the stimulus frequency was extracted by Fourier analysis. Images were smoothed by a 5 × 5 low-pass Gaussian filter and the binocular region of interest (ROI) was defined as the 70% of pixels with the highest intensity in the ipsilateral eye map (MATLAB, Mathworks, Natick, MA). The average number of pixels activated by the full visual field stimulus was calculated as a relative measure of V1 size. The ocular dominance value of each pixel in the binocular region was calculated as (contra-ipsi)/(contra+ipsi) and averaged to obtain the ODI.

#### Statistics

Data were analyzed with 2-tailed unpaired *t*-tests, Mann-Whitney tests, 2-way ANOVAs (±repeated measures) with Holm-Sidak posthoc analysis, or Kruskal-Wallis with Dunn’s posthoc analysis, as indicated in the figure and table legends (GraphPad Prism, San Diego, CA). *p <* 0.05 was considered significant. In cases where data were not normally distributed (Kolmogorov-Smirnov normality test), nonparametric tests were used. The Rout test (Q = 0.1% was used to identify outliers, which were then excluded from statistical analysis. Statistical analysis was performed using data from each cell (whole-cell patch clamp recordings) or each animal (polysomnography, optical imaging). Sample size is displayed in the figures as (number of cells, number of animals) or as number of animals only. Lines and error bars in all figure dot plots indicate mean and SEM.
