# Daily oscillation of the excitation/inhibition ratio is disrupted in two mouse models of autism

**DOI:** 10.1016/j.isci.2025.111917

**Published:** 2025-02-04

**Authors:** Michelle C.D. Bridi, Nancy Luo, Grace Kim, Benjamin J. Menarchek, Rachel A. Lee, Bryan Rodriguez, Daniel Severin, Cristian Moreno, Altagracia Contreras, Christian Wesselborg, Caroline O’Ferrall, Ruchit Patel, Sarah Bertrand, Sujatha Kannan, Alfredo Kirkwood

## Main text

(iScience *28*, 111494; January 17, 2025)

Due to a production oversight, authors Cristian Moreno and Altagracia Contreras appeared in the wrong order in the author list. This has now been corrected online. We apologize for any inconvenience.

