# Supplementary material for: Daily oscillation of the excitation/inhibition ratio is disrupted in two mouse models of autism
Source: iScience. 2024 Dec 5;28(1):111494. doi: 10.1016/j.isci.2024.111494 (PMC11754079; doi:10.1016/j.isci.2024.111494)
Supplement: Document S1. Figures S1–S4 and Tables S1–S4 [file mmc1.pdf]

iScience, Volume *28*

Supplemental information

Daily oscillation of the excitation/inhibition ratio is disrupted in two mouse models of autism

Michelle C.D. Bridi, Nancy Luo, Grace Kim, Benjamin J. Menarchek, Rachel A. Lee, Bryan Rodriguez, Daniel Severin, Cristian Moreno, Altagracia Contreras, Christian Wesselborg, Caroline O'Ferrall, Ruchit Patel, Sarah Bertrand, Sujatha Kannan, and Alfredo Kirkwood

Supplementary Figures.

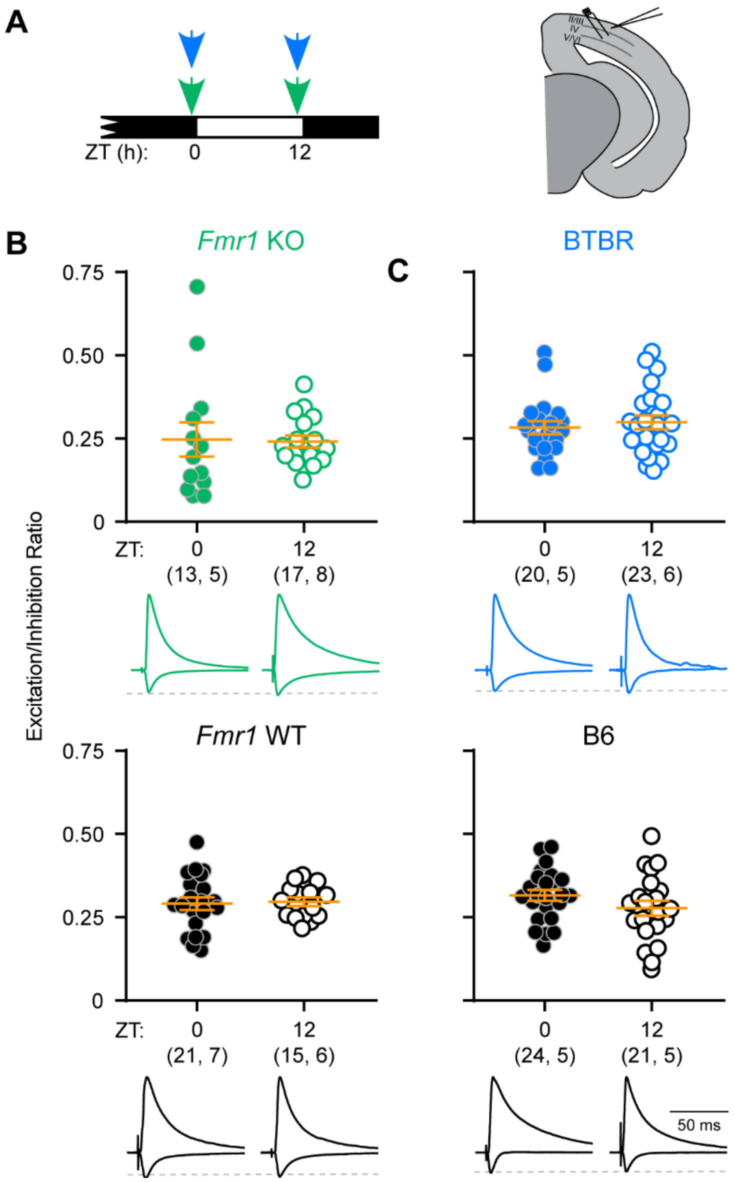

Supplementary Figure 1 (Related to Figure 1 and Tables S1, S2). The layer 4-2/3 E/I ratio is not affected by time of day. (A) *Fmr1* WT, *Fmr1* KO, B6, and BTBR mice were sacrificed at two times of day and acute brain slices containing V1 were collected for whole-cell patch clamp recordings of layer 2/3 pyramidal neurons in response to layer 4 stimulation. (B, C) The E/I ratio was not different between ZT0 and ZT12 in *Fmr1* KO, BTBR, or WT mice (2-tailed *t* tests within genotype; See Table S1). When each line was compared to its WT control using 2-way ANOVAs, no significant main effects of time, genotype, or time × genotype interaction were observed (See Table S1). Sample size is indicated as (cells, mice). Error bars indicate mean±SEM. Example traces show the inhibitory response (upward deflection) and excitatory response (downward deflection) in the same cell and are normalized to peak inhibitory response.

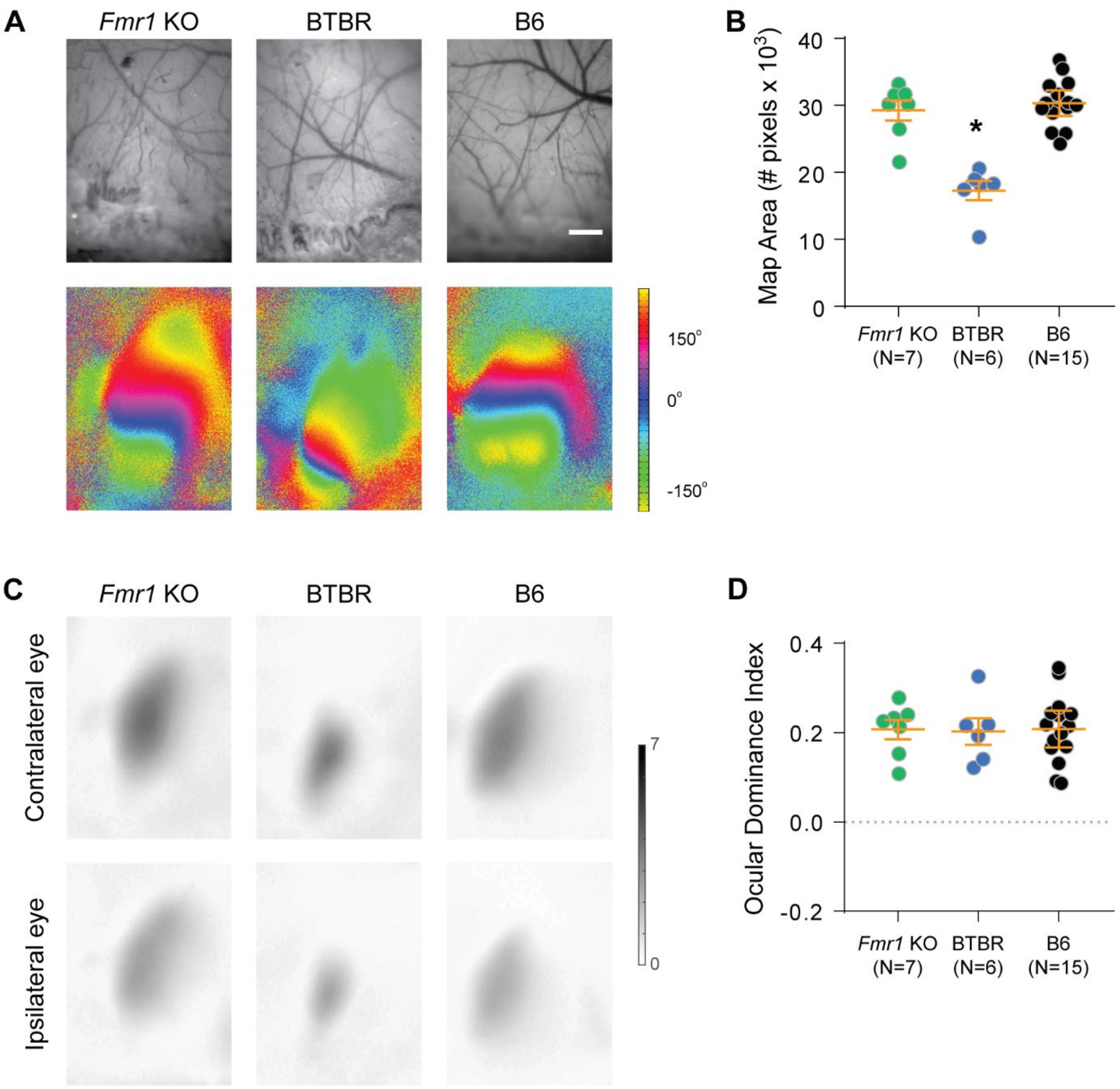

Supplementary Figure 2 (Related to Figure 1). V1 is functional and expresses normal ocular dominance bias in *Fmr1* KO and BTBR mice. (A) Example images of the cortical surface vasculature (top) and retinotopic maps (bottom) obtained by optical imaging of the intrinsic cortical signal while presenting a visual stimulus to the entire visual field of both eyes simultaneously. V1 of both mouse lines was functional and displayed retinotopic organization. Scale bar: 1mm. **(B)** V1 size was normal in *Fmr1* KO mice but smaller in BTBR mice, compared with WT controls (shaded gray region indicates mean ± 95% CI of B6 mice). Kruskal-Wallis ANOVA on ranks P=0.0011; *Fmr1* KO P>0.999, BTBR P=0.0007, Dunn's post-hoc test vs. B6). **(C)** Example images showing the magnitude of response to each eye during presentation of a visual stimulus to the binocular visual field. **(D)** The ocular dominance index in binocular V1 was normal in both *Fmr1* KO and BTBR mice, compared to WT controls (shaded gray region indicates mean ± 95% CI of B6 mice). ANOVA $F_{(2, 25)}$=0.014, P=0.986. Sample size is indicated as (# mice) and error bars represent SEM.

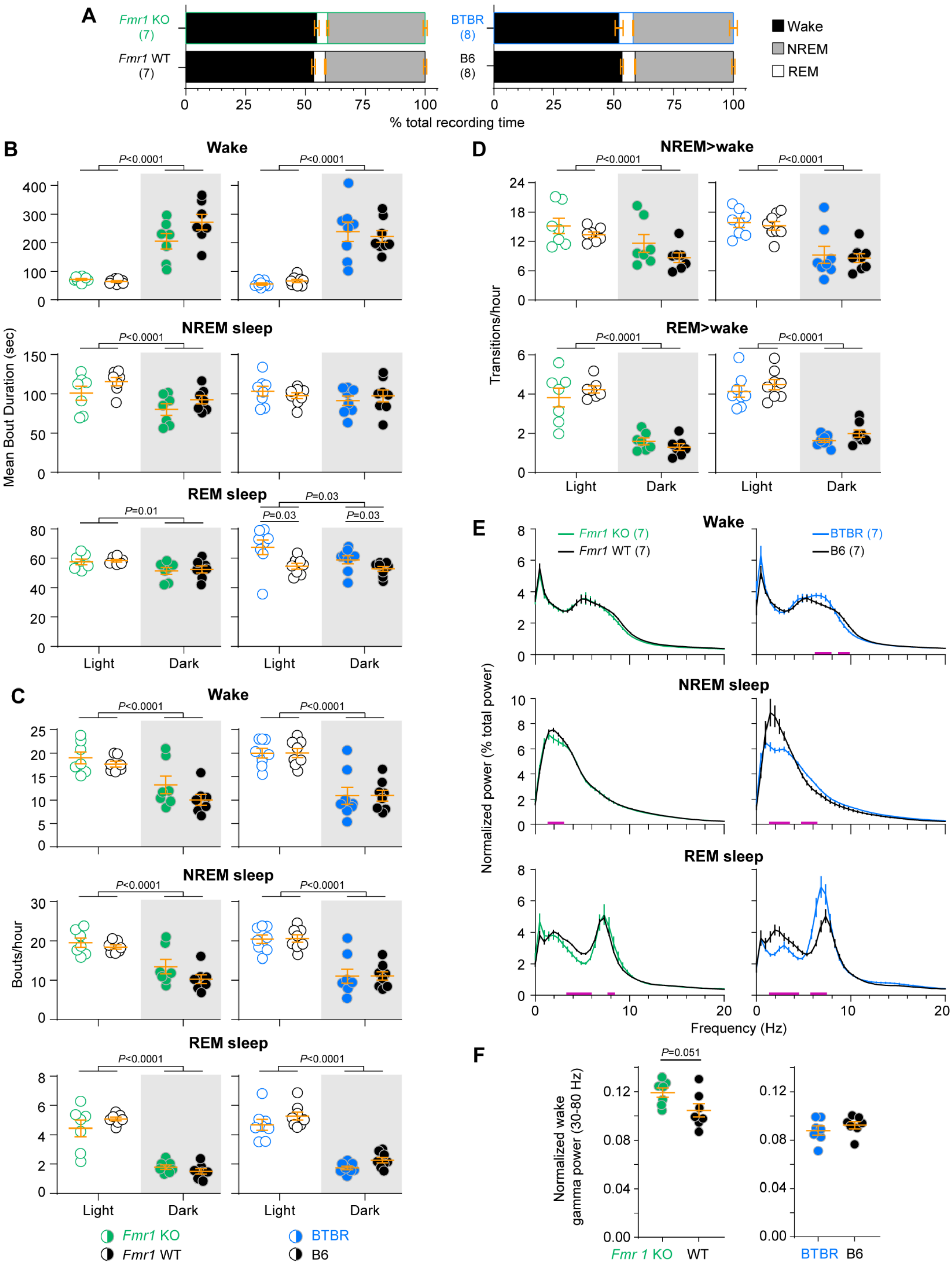

A
Fmr1 KO (7)
Fmr1 WT (7)
BTBR (8)
B6 (8)
Wake
NREM
REM
% total recording time
B
Wake
NREM sleep
REM sleep
Mean Bout Duration (sec)
P<0.0001
P=0.01
P=0.03
Light
Dark
C
Bouts/hour
D
NREM>wake
REM>wake
Transitions/hour
E
Fmr1 KO (7)
Fmr1 WT (7)
BTBR (7)
B6 (7)
Normalized power (% total power)
Frequency (Hz)
F
Normalized wake gamma power (30-80 Hz)
P=0.051
Fmr 1 KO
WT
BTBR
B6

Supplementary Figure 3 (Related to Figure 2 and Table S3). Sleep architecture and EEG power in ASD model mice. **(A)** Overall amounts of wake, NREM sleep, and REM sleep did not differ between *Fmr1* KO and WT mice, or between BTBR and B6 mice (2-tailed *t* test $P>0.05$; Supplementary Table 3). **(B)** Sleep/wake bout durations did not differ between *Fmr1* KO and WT mice. Both *Fmr1* KO and WT mice had shorter wake and longer sleep bouts during the light phase. BTBR mice, on the other hand, showed subtle changes in sleep architecture. REM sleep bouts were significantly longer in both the light and dark phases compared to B6. Both genotypes had shorter wake and longer REM bouts during the light phase. *P* values indicate significance on 2-way repeated measures ANOVAs; See Table S3. **(C,D)** All arousal states were more fragmented (more bouts and sleep-wake transitions) during the light phase than during than during the dark phase. No main effects of genotype or genotype × time interactions were observed. *P* values indicate significance on 2-way repeated measures ANOVAs (See Table S3). **(E)** Power spectra were calculated separately for wake, NREM sleep, and REM sleep arousal states and normalized to total spectral power (0.5-80 Hz) within that state. Data were compared using 2-way ANOVAs with genotype and frequency as main factors (See Table S3). Magenta lines indicate the frequencies at which genotypes are significantly different (Holm-Sidak post-hoc test $P<0.05$). **(F)** Wake power in the gamma range showed a strong trend toward higher power in *Fmr1* KO than WT controls, but no difference between B6 and BTBR mice (2-tailed *t* test; Table S3). For all panels, sample size is indicated as (mice) and bars represent mean ± SEM.

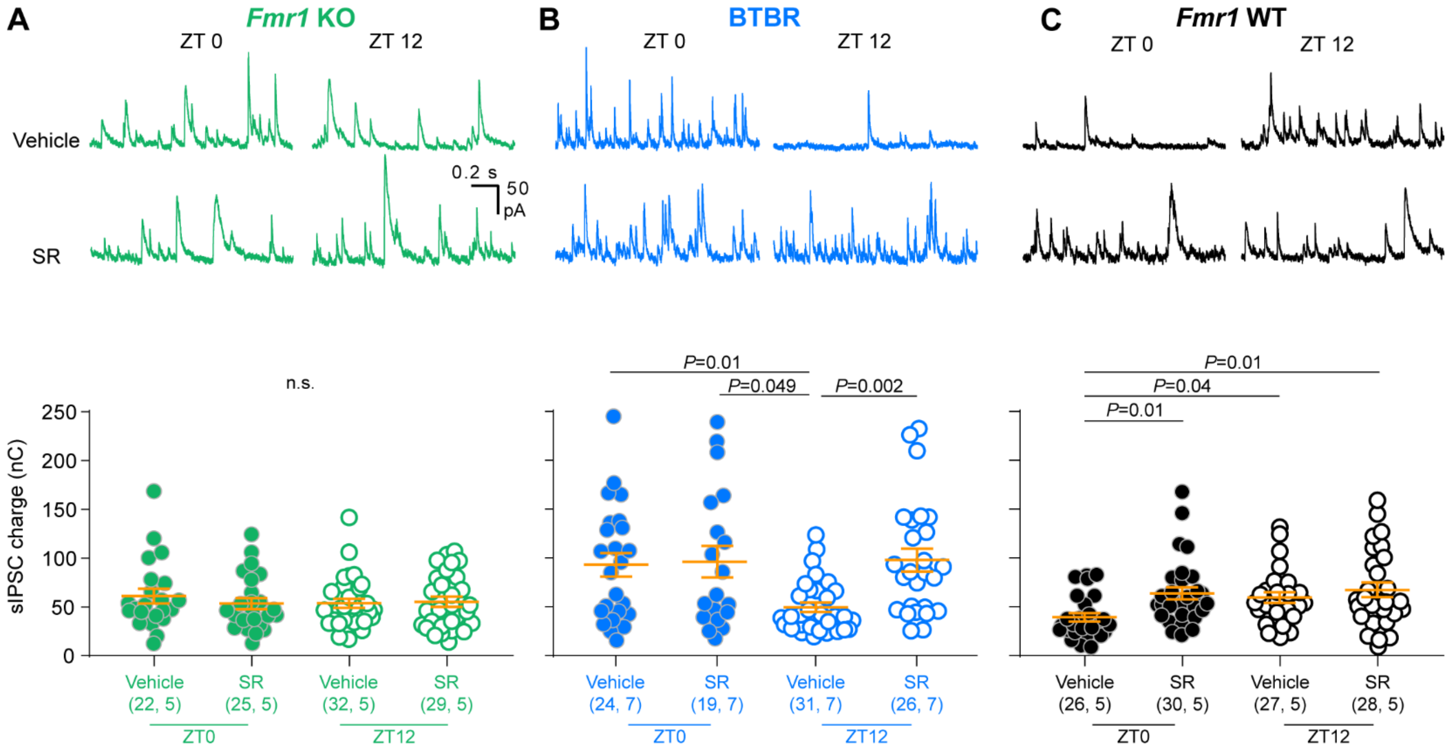

Supplementary Figure 4 (Related to Figure 5). eCB signaling is flattened in *Fmr1* KO and timing is altered in BTBR mice. Acute brain slices containing V1 were obtained at ZT0 and ZT12, and sIPSCs in layer 2/3 pyramidal cells were recorded in the presence and absence of the eCB inverse agonist SR (10μM). (A) Example traces (top) and quantification (bottom) of sIPSCs from *Fmr1* KO mice. SR does not elevate inhibitory transmission at either time point, consistent with low eCB signaling across the 24h day. Kruskal-Wallis ANOVA on ranks *H*=0.64, *P*=0.89. (B) Example traces (top) and quantification (bottom) of sIPSCs from BTBR mice. SR enhances inhibitory transmission only in slices collected at the end of the light phase. Kruskal-Wallis ANOVA on ranks *H*=16.4, *P*=0.001. Dunn's post-hoc test *P* values are indicated. (C) Example traces (top) and quantification (bottom) of sIPSCs from *Fmr1* WT mice. SR enhances inhibitory transmission only in slices obtained at the end of the dark phase. Kruskal-Wallis ANOVA on ranks *H*=12.9, *P*=0.005. Dunn's post-hoc test *P* values are indicated. (A-C) Sample size is indicated as (cells, mice) and bars represent mean ± SEM.

Supplementary Tables.

Supplementary Table 1 (Related to Figures 1 and S1). Excitatory/inhibitory synaptic transmission test statistics. Time-of-day changes within each genotype were detected using 1-way ANOVAs and *t* tests (BTBR/B6). For each mouse model, 2-way ANOVAs with genotype (ASD-related vs WT) and time of day as factors were also conducted. *Fmr1* lateral E/I ratio data were pooled within the dark (ZT0, 18) and light (ZT6, 12) phases for the 2-way ANOVA only. Data figures corresponding to each test are indicated. *t*: Student's *t* test; *U*: Mann-Whitney U test; *H*: Kruskal-Wallis ANOVA on Ranks; *F*: 1- or 2-way ANOVA.

			Fmr1 KO/WT		BTBR/B6	
	figure	factor	test statistic	*P*	test statistic	*P*
Lateral E/I ratio	1	Time of Day (Fmr1 KO/BTBR)	$H_{(3, 102)}$=1.62	0.65	$t_{(45)}$=2.64	**0.01***
		Time of Day (WT control)	$F_{(3, 94)}$=6.67	**0.0004***	$t_{(45)}$=2.93	**0.006***
		Genotype	$F_{(1, 200)}$=1.9	0.17	$F_{(1, 86)}$=0.6	0.44
		Time of Day	$F_{(1, 200)}$=6.8	**0.01***	$F_{(1, 86)}$=1.5	0.23
		Interaction	$F_{(1, 200)}$=10.5	**0.001***	$F_{(1, 86)}$=15.6	**0.0002***
Vertical E/I ratio	S1	Time of Day (Fmr1 KO/BTBR)	$t_{(28)}$=0.13	0.90	$t_{(41)}$=0.56	0.58
		Time of Day (WT control)	$t_{(34)}$=0.22	0.82	$t_{(43)}$=1.44	0.16
		Genotype	$F_{(1, 62)}$=3.4	0.07	$F_{(1, 84)}$=0.08	0.78
		Time of Day	$F_{(1, 62)}$=0.0002	0.99	$F_{(1, 84)}$=0.32	0.57
		Interaction	$F_{(1, 62)}$=0.05	0.82	$F_{(1, 84)}$=2.0	0.17
mEPSC frequency	3, 4	Time of day (Fmr1 KO/BTBR)	*U*=613	0.35	*U*=322	**0.016***
		Time of day (WT control)	*U*=269	**0.001***	$t_{(67)}$=2.53	**0.014***
		Genotype	$F_{(1, 135)}$=14.5	**0.0002***	$F_{(1, 128)}$=0.4	0.51
		Time of Day	$F_{(1, 135)}$=2.6	0.11	$F_{(1, 128)}$=2E-7	0.9996
		Interaction	$F_{(1, 135)}$=9.3	**0.003***	$F_{(1, 128)}$=14.1	**0.0003***
mIPSC frequency	3, 4	Time of day (Fmr1 KO/BTBR)	*U*=596	0.93	*U*=278	**0.026***
		Time of day (WT/B6)	$t_{(51)}$=2.69	**0.0098***	*U*=464	**0.017***
		Genotype	$F_{(1, 119)}$=0.4	0.51	$F_{(1, 128)}$=7.6	**0.007***
		Time of Day	$F_{(1, 119)}$=4.6	**0.03***	$F_{(1, 128)}$=1.3	0.26
		Interaction	$F_{(1, 119)}$=1.5	0.22	$F_{(1, 128)}$=13.1	**0.0004***

Supplementary table 2 (Related to Figures 1 and S1). **Mean±SEM of E/I ratio values reported in Figure 1 and Supplementary Figure 1**. *P<0.05 compared to ZT0 values within the same genotype; see Supplementary table 1 for statistical comparisons.

		Fmr1 KO	*Fmr1* WT	BTBR	B6
lateral	ZT0	0.23±0.024	0.30±0.011	0.28±0.011	0.36±0.021
	ZT6	0.25±0.024	**0.20±0.016***		
	ZT12	0.23±0.012	**0.22±0.015***	**0.32±0.015***	**0.26±0.023***
	ZT18	0.23±0.020	0.29±0.027		
Vertical	ZT0	0.25±0.052	0.29±0.019	0.28±0.020	0.32±0.016
	ZT12	0.24±0.018	0.30±0.013	0.30±0.021	0.28±0.022

Supplementary Table 3 (Related to Figures 2 and S3). Test statistics for sleep analyses. *F*: 2-way RM ANOVA; *t*: 2-tailed Student's *t* test, *U*: Mann-Whitney *U* test.

			Fmr1 KO/WT		BTBR/B6	
	figure	factor	test statistic	*P*	test statistic	*P*
% Wake (1h bins)	2	Genotype	$F_{(1, 12)}$=3.43	0.09	$F_{(1, 14)}$=0.72	0.41
		Time of Day	$F_{(23, 276)}$=25.9	**<0.0001***	$F_{(23, 322)}$=17.2	**<0.0001***
		Interaction	$F_{(23, 276)}$=0.88	0.63	$F_{(23, 322)}$=1.57	**0.048***
%NREM (1h bins)	2	Genotype	$F_{(1, 12)}$=2.80	0.12	$F_{(1, 14)}$=0.48	0.50
		Time of Day	$F_{(23, 276)}$=24.4	**<0.0001***	$F_{(23, 322)}$=16.2	**<0.0001***
		Interaction	$F_{(23, 276)}$=0.88	0.63	$F_{(23, 322)}$=1.52	0.06
%REM (1h bins)	2	Genotype	$F_{(1, 12)}$=0.86	0.37	$F_{(1, 14)}$=0.54	0.47
		Time of Day	$F_{(23, 276)}$=22.1	**<0.0001***	$F_{(23, 322)}$=19.8	**<0.0001***
		Interaction	$F_{(23, 276)}$=0.76	0.78	$F_{(23, 322)}$=2.05	**0.004***
%Wake (light vs dark)	2	Genotype	$F_{(1, 12)}$=3.4	0.09	$F_{(1,14)}$=0.83	0.38
		Time of Day	$F_{(1, 12)}$=153.0	**<0.0001***	$F_{(1, 14)}$=259.3	**<0.0001***
		Interaction	$F_{(1, 12)}$=3.1	0.10	$F_{(1, 14)}$=2.5	0.14
%NREM (light vs dark)	2	Genotype	$F_{(1, 12)}$=2.8	0.12	$F_{(1, 14)}$=0.49	0.5
		Time of Day	$F_{(1, 12)}$=162.9	**<0.0001***	$F_{(1, 14)}$=232	**<0.0001***
		Interaction	$F_{(1,12)}$=3.3	0.09	$F_{(1, 14)}$=2.0	0.18
%REM (light vs dark)	2	Genotype	$F_{(1, 12)}$=0.86	0.37	$F_{(1, 14)}$=0.54	0.47
		Time of Day	$F_{(1, 12)}$=85.6	**<0.0001***	$F_{(1, 14)}$=342.5	**<0.0001***
		Interaction	$F_{(1,12)}$=1.8	0.20	$F_{(1, 14)}$=4.93	**0.04***
%Wake (total)	S3A	Genotype	$t_{(12)}$=1.1	0.3	$t_{(14)}$=0.85	0.41
%NREM (total)	S3A	Genotype	$t_{(12)}$=0.96	0.36	$t_{(14)}$=0.69	0.50
% REM (total)	S3A	Genotype	$U_{(12)}$=22	0.80	$t_{(14)}$=0.73	0.48
Wake Bout duration	S3B	Genotype	$F_{(1,12)}$=2.7	0.13	$F_{(1, 14)}$=0.03	0.87
		Time of Day	$F_{(1,12)}$=71.1	**<0.0001***	$F_{(1, 14)}$=71.8	**<0.0001***
		Interaction	$F_{(1,12)}$=3.36	0.09	$F_{(1, 14)}$=0.51	0.49
NREM Bout duration	S3B	Genotype	$F_{(1,12)}$=2.14	0.17	$F_{(1, 14)}$=0.001	0.97
		Time of Day	$F_{(1,12)}$=61.8	**<0.0001***	$F_{(1, 14)}$=2.62	0.13
		Interaction	$F_{(1,12)}$=0.20	0.67	$F_{(1, 14)}$=2.42	0.14
REM Bout duration	S3B	Genotype	$F_{(1,12)}$=0.22	0.65	$F_{(1, 14)}$=6.05	**0.03***
		Time of Day	$F_{(1,12)}$=9.4	**0.01***	$F_{(1, 14)}$=5.92	**0.03***
		Interaction	$F_{(1,12)}$=0.004	0.95	$F_{(1, 14)}$=2.54	0.13
Wake bouts/hour	S3C	Genotype	$F_{(1,12)}$=1.72	0.21	$F_{(1, 14)}$=0.0004	0.98
		Time of Day	$F_{(1,12)}$=117.7	**<0.0001***	$F_{(1, 14)}$=117.2	**<0.0001***
		Interaction	$F_{(1,12)}$=2.25	0.16	$F_{(1, 14)}$=1.06E-6	0.999
NREM bouts/hour	S3C	Genotype	$F_{(1, 12)}$=1.66	0.22	$F_{(1, 14)}$=0.005	0.94
		Time of Day	$F_{(1, 12)}$=127.8	**<0.0001***	$F_{(1, 14)}$=135.4	**<0.0001***
		Interaction	$F_{(1, 12)}$=2.65	0.13	$F_{(1, 14)}$=0.003	0.96
REM bouts/hour	S3C	Genotype	$F_{(1,12)}$=0.31	0.59	$F_{(1, 14)}$=3.34	0.09
		Time of Day	$F_{(1,12)}$=92.6	**<0.0001***	$F_{(1, 14)}$=270.5	**<0.0001***
		Interaction	$F_{(1, 12)}$=2.03	0.18	$F_{(1, 14)}$=0.03	0.86
NREM>wake transitions	S3D	Genotype	$F_{(1, 12)}$=1.63	0.23	$F_{(1, 14)}$=0.21	0.66
		Time of Day	$F_{(1, 12)}$=107.7	**<0.0001***	$F_{(1, 14)}$=57.8	**<0.0001***
		Interaction	$F_{(1, 12)}$=1.96	0.19	$F_{(1, 14)}$=2.5E-5	0.996

REM>wake transitions	S3D	Genotype	$F_{(1, 12)}$=0.05	0.83	$F_{(1, 14)}$=1.71	0.21
		Time of Day	$F_{(1, 12)}$=75.7	**<0.0001***	$F_{(1, 14)}$=246.1	**<0.0001***
		Interaction	$F_{(1, 12)}$=1.45	0.25	$F_{(1, 14)}$=0.001	0.97
Wake power	S3E	Genotype	$F_{(1, 1932)}$=0.13	0.72	$F_{(1, 1932)}$=0.37	0.54
		Frequency	$F_{(160, 1932)}$=429.9	**<0.0001***	$F_{(160, 1932)}$=549.4	**<0.0001***
		Interaction	$F_{(160, 1932)}$=0.49	<0.999	$F_{(160, 1932)}$=1.73	**<0.0001***
NREM power	S3E	Genotype	$F_{(1, 1932)}$=0.24	0.62	$F_{(1, 1932)}$=0.02	0.88
		Frequency	$F_{(160, 1932)}$=1770	**<0.0001***	$F_{(160, 1932)}$=372.1	**<0.0001***
		Interaction	$F_{(160, 1932)}$=1.58	**<0.0001***	$F_{(160, 1932)}$=6.47	**<0.0001***
REM power	S3E	Genotype	$F_{(1, 1932)}$=0.26	0.61	$F_{(1, 1932)}$=0.04	0.84
		Frequency	$F_{(160, 1932)}$=277.4	**<0.0001***	$F_{(160, 1932)}$=223.0	**<0.0001***
		Interaction	$F_{(160, 1932)}$=1.49	**0.0001***	$F_{(160, 1932)}$=6.65	**<0.0001***
Wake gamma power	S3F	Genotype	$t_{(12)}$=2.17	0.051	$t_{(12)}$=0.92	0.38

Supplementary Table 4 (Related to Figures 3, 4). mE/IPSC characteristics. Groups were compared using *t* or Mann-Whitney *U* tests, as indicated. Data are shown as mean ± SEM.

		Amplitude	Rise	Decay	N
Fmr1 KO/WT mEPSCs	KO ZT0	13.20 ± 0.4	0.79 ± 0.01	2.53 ± 0.05	38 , 5
	KO ZT12	13.67 ± 0.3	0.78 ± 0.02	2.63 ± 0.06	37 , 6
	test statistic	*U*=579	*U*=690	*U*=592.5	
	P	0.19	0.89	0.24	
	WT ZT0	12.58 ± 0.3	0.77 ± 0.02	2.60 ± 0.05	34 , 5
	WT ZT12	13.09 ± 0.4	0.77 ± 0.02	2.52 ± 0.06	30 , 5
	test statistic	*U*=445	$t_{(62)}$=0.23	*U*=415	
	P	0.39	0.82	0.2	
Fmr1 KO/WT mIPSCs	KO ZT0	46.01 ± 1.7	0.65 ± 0.03	5.58 ± 0.20	39 , 6
	KO ZT12	42.60 ± 2.1	0.66 ± 0.03	6.37 ± 0.28	31 , 6
	test statistic	$t_{(68)}$=1.26	*U*=576	$t_{(68)}$=2.36	
	P	0.21	0.74	**0.02***	
	WT ZT0	38.80 ± 2.8	0.75 ± 0.05	6.00 ± 0.33	27 , 6
	WT ZT12	40.47 ± 0.2	0.69 ± 0.03	5.64 ± 0.20	26 , 6
	test statistic	$t_{(51)}$=0.46	*U*=330.5	$t_{(51)}$=0.94	
	P	0.65	0.73	0.35	
BTBR/B6 mEPSCs	BTBR ZT0	13.6 ± 0.6	0.8 ± 0.03	2.7 ± 0.1	32 , 6
	BTBR ZT12	12.8 ± 0.5	0.7 ± 0.02	2.5 ± 0.1	31 , 5
	test statistic	$t_{(61)}$=1.08	$t_{(61)}$=1.49	$t_{(61)}$=1.60	
	P	0.28	0.14	0.11	
	B6 ZT0	13.7 ± 0.5	0.8 ± 0.03	2.8 ± 0.1	38 , 10
	B6 ZT12	13.6 ± 0.4	0.8 ± 0.03	3.1 ± 0.1	31 , 9
	test statistic	$t_{(67)}$=0.91	*U*=511	*U*=461.5	
	P	0.11	0.35	0.12	
BTBR/B6 mIPSCs	BTBR ZT0	45.6 ± 2.0	0.5 ± 0.01	3.8 ± 0.1	29 , 6
	BTBR ZT12	49.0 ± 3.2	0.5 ± 0.02	4.0 ± 0.2	29 , 6
	test statistic	$t_{(56)}$=0.93	$t_{(56)}$=0.27	$t_{(56)}$=0.76	
	P	0.36	0.79	0.45	
	B6 ZT0	45.2 ± 2.1	0.5 ± 0.02	4.2 ± 0.2	36 , 6
	B6 ZT12	40.6 ± 1.8	0.6 ± 0.02	4.4 ± 0.2	38 , 6
	test statistic	$t_{(72)}$=1.71	$t_{(72)}$=1.82	*U*=648	
	P	0.09	0.07	0.70	
